# Pragmatics to Reveal Intent in Social Media Peer Interactions: Mixed Methods Study

**DOI:** 10.2196/32167

**Published:** 2021-11-17

**Authors:** Tavleen Singh, Sofia Olivares, Trevor Cohen, Nathan Cobb, Jing Wang, Amy Franklin, Sahiti Myneni

**Affiliations:** 1 School of Biomedical Informatics University of Texas Health Science Center Houston, TX United States; 2 Biomedical Informatics and Medical Education University of Washington Seattle, WA United States; 3 Georgetown University Medical Center Washington, DC United States; 4 Florida State University College of Nursing Tallahassee, FL United States

**Keywords:** online health communities, diabetes self-management, tobacco cessation, speech acts, behavior change, communication themes

## Abstract

**Background:**

Online health communities (OHCs) have emerged as the leading venues for behavior change and health-related information seeking. The soul and success of these digital platforms lie in their ability to foster social togetherness and a sense of community by providing personalized support. However, we have a minimal understanding of how conversational posts in these settings lead to collaborative societies and ultimately result in positive health changes through social influence.

**Objective:**

Our objective is to develop a content-specific and intent-sensitive methodological framework for analyzing peer interactions in OHCs.

**Methods:**

We developed and applied a mixed-methods approach to understand the manifestation of expressions in peer interactions in OHCs. We applied our approach to describe online social dialogue in the context of two online communities, QuitNet (QN) and the American Diabetes Association (ADA) support community. A total of 3011 randomly selected peer interactions (n=2005 from QN, n=1006 from ADA) were analyzed. Specifically, we conducted thematic analysis to characterize communication content and linguistic expressions (speech acts) embedded within the two data sets. We also developed an empirical user persona based on their engagement levels and behavior profiles. Further, we examined the association between speech acts and communication themes across observed tiers of user engagement and self-reported behavior profiles using the chi-square test or the Fisher test.

**Results:**

Although social support, the most prevalent communication theme in both communities, was expressed in several subtle manners, the prevalence of emotions was higher in the tobacco cessation community and assertions were higher in the diabetes self-management (DSM) community. Specific communication theme-speech act relationships were revealed, such as the social support theme was significantly associated (*P*<.05) with 9 speech acts from a total of 10 speech acts (ie, assertion, commissive, declarative, desire, directive, expressive, question, stance, and statement) within the QN community. Only four speech acts (ie, commissive, emotion, expressive, and stance) were significantly associated (*P*<.05) with the social support theme in the ADA community. The speech acts were also significantly associated with the users’ abstinence status within the QN community and with the users’ lifestyle status within the ADA community (*P*<.05).

**Conclusions:**

Such an overlay of communication intent implicit in online peer interactions alongside content-specific theory-linked characterizations of social media discourse can inform the development of effective digital health technologies in the field of health promotion and behavior change. Our analysis revealed a rich gradient of expressions across a standardized thematic vocabulary, with a distinct variation in emotional and informational needs, depending on the behavioral and disease management profiles within and across the communities. This signifies the need and opportunities for coupling pragmatic messaging in digital therapeutics and care management pathways for personalized support.

## Introduction

Lifestyle risk factors, such as tobacco use, poor diet, and physical inactivity, play an essential role in chronic disease management [1]. These health risk behaviors, albeit modifiable, result in a large number of premature deaths in the United States [2,3]. Although there are illness-specific self-management tasks, the adoption and maintenance of health behaviors are core tasks for the management of multiple chronic diseases, such as cancer, diabetes, and cardiovascular conditions [4-6]. Numerous interventions and public health campaigns have been developed to help patients incorporate new behaviors (eg, adherence to medication regimens) [7] and modify existing risky behaviors (eg, smoking cessation) [8,9] to prevent and manage chronic diseases and promote well-being. However, adherence to healthy behaviors requires significant support that targets individualistic factors and environmental influences for long time intervals [10,11].

Online health communities (OHCs) have gained popularity as venues for behavior change [12-17]. These platforms provide novel opportunities to understand complex relationships among individual actions and environmental influences in health behavior change. With the onset of mobility and connectivity in the communication sector, messages exchanged in health-related online communities reflect the intricacies of human health behavior as experienced in real time at the individual, community, and societal levels [18]. The majority of studies that have examined communication content in online communities have been limited to (1) content-agnostic analysis, such as identifying the structural characteristics of online social networks to understand how such differences might impact smoking-related behaviors [19,20]; (2) content-rich analysis, such as identifying topics, themes, and opinions from diabetes-related conversations [21,22] or capturing sentiments of individuals toward alternative smoking products [23]; and (3) content-inclusive social network analysis, such as combining content-dependent attributes with social network ties to analyze what content is being exchanged in a social tie to explain how social relationships influence behavior change [24,25]. Although several studies on health-related online communities have used manual and automated methods to analyze the content of communication, few studies have focused on the latent intent of the communication, thus ignoring the essential context relevant to health-related decision making.

In this paper, using the concepts drawn from pragmatics [26], we focus on the context of language in use, in addition to the form or content of health-related online exchanges. Pragmatic analyses enable the characterization of communication intent. Social media communication may express beliefs, ask questions, and direct another person to act. In addition to the content of the message, the context of how it is said and delivered layers further meaning to the exchange. Although pragmatics capture many aspects of semiotics (inclusive of semantics and syntax) in the transmission of a message, we narrow our scope to the speech acts prevalent in threaded discussions. Language can not only state ideas but also accomplish action through speech acts [27-29]. According to speech act theory, which was introduced by Austin [27] and later developed by Searle [28,29], speech acts capture the range of impact, or force, expressed within messages, including promises, warnings, proclamations, and statements [28,29]. Previous work on speech act analysis within online text-based communication has been applied to synchronous conversations, such as away messages on instant messengers [30], or asynchronous conversations, such as emails [31], status messages on Facebook [32,33], consumer reviews [34], forum posts [35], tweets [36,37], and emojis in WhatsApp chats [38]. Such analysis has revealed how individuals make statements, ask questions, offer suggestions, comment, and produce other speech acts that can be used to describe the strength of overall consumer sentiments through understanding implicit expressions and discourse patterns.

Although the majority of earlier works using pragmatics are in the general domain, in this paper we propose a methodological framework using speech act theory [27-29] and apply it to the messages exchanged in OHCs. Leveraging our prior work [22,39], which focused on the extraction of conversational themes and mapping of health behavior theory, we describe the relationships between theory-linked communication themes embedded in health-related peer interactions and their manifestation as individuals attempt behavior modification and self-management of chronic conditions. To ensure generalizability, our methodological framework was applied in the context of two different OHCs: (1) one for tobacco cessation and (2) another for DSM. We chose these two domains because existing research has established the influence of social relationships on risky health behaviors (eg, tobacco cessation) and lifestyle factors (eg, diet, physical activity, medication use, and self-monitoring of blood glucose) that impact type 2 diabetes mellitus (T2DM)-related health outcomes; for example, an individual is more likely to comply with health-related goals and adhere to preventive practices, provided their social ties also engage in similar behaviors by changing their intrapersonal beliefs, attitudes, or knowledge [40-43]. Through this analysis, we aim to address the following research questions:

(1) How are communication content and intent expressed in OHCs for behavior change and chronic disease management?

(2) How is communication intent associated with self-reported user behavior and observed user engagement in an OHC?

## Methods

**Figure 1:**
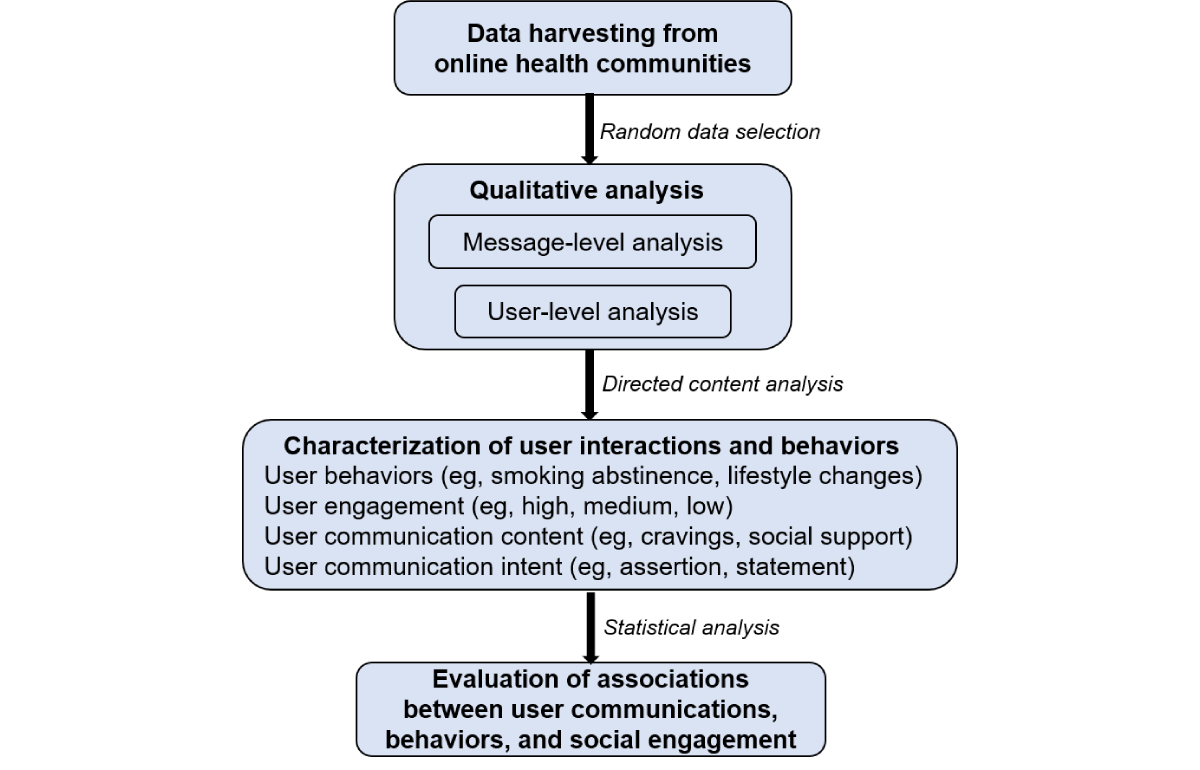
Outline of the methodological framework of the study.

Figure 1 describes the important components of our mixed-methods approach, which are explained in more detail in subsequent sections.

### Data Set Selection

The proposed research was conducted in the context of two different OHCs: one for tobacco cessation and another for DSM.

*QuitNet* (QN) is one of the largest OHCs promoting tobacco cessation amongst its users [44]. The users of this community are usually smokers willing to quit or ex-smokers willing to stay abstinent. Initial studies have shown a strong correlation of an individual’s participation in QN with abstinence compared to individuals who do not participate in such communities [45]. The data set used in this study was drawn from a previously studied quality improvement database spanning from 2000 to 2015, consisting of nearly 2.46 million peer interactions organized into 289,456 unique threads exchanged by 64,884 unique users. Using the inbuilt sample() function of the random module in Python, we randomly selected a subset of 2005 messages from this data set, organized into 132 unique threads posted by 884 unique community users.

The *American Diabetes Association* (ADA) support community is an online support group for individuals who have diabetes (type 1, type 2, or prediabetes) to engage with their peers as well as their caregivers [46]. The community users interact with one another on a wide variety of topics ranging from medication use, diet, and physical activity to daily monitoring of blood glucose levels. Even though the outcomes among type 1 diabetes mellitus (T1DM), T2DM, or prediabetes are impacted by behaviors (eg, lifestyle, medication use, self-monitoring of blood glucose) that can be heavily influenced by an individual’s social infrastructure, for this research, we focused on interactions related to T2DM. The data set spanned from 2015 to 2018, consisting of nearly 58,965 peer interactions specific to T2DM organized into 5829 unique topics posted by 1909 users. Using the inbuilt sample() function of the random module in Python, we randomly selected a subset of 1006 messages from this data set, organized into 99 unique topics posted by 141 unique community users.

Based on our prior research that focused on the qualitative analysis of digital communication from these OHCs [22,39], we expanded our data sets for this study in order to attain thematic saturation and a justifiable data set size amenable to manual analysis. Further, we selected the given size ranges for the two data sets in order to create a gold-standard annotated data set that can be efficiently used for downstream automated text mining techniques.

### Qualitative Analysis

#### Message-Level Content and Intent Characterization

We conducted an in-depth qualitative analysis, in which we used directed content analysis [47] and discourse analysis [48] techniques, of a random sample of 3011 messages from QN and ADA online communities. Based on a modified version of Searle's taxonomy of speech acts [28,29], we classified the messages using discourse analysis into 10 categories, as described in Multimedia Appendix 1. By combining the speech acts (how) with the content (what) of these communications, we can understand how the community users accomplish their common goals, such as tobacco cessation or diabetes control—that is, how, using online exchanges, do these communities shape their identity, support their users, and accomplish the goal of helping individuals sustain positive health changes effectively and efficiently. Further, we used the coding schema provided in Multimedia Appendix 2 to map speech acts to inductively derived, theoretically linked communication themes [22,46,49]. Communication themes capture the essence or meaning of the conversation (which essentially pertains to communication content). They are obtained through the aggregation of behavioral and cognitive constructs from multiple behavior change theories [22,46,49]. The communication theme codes were inductively derived using grounded theory techniques in our prior work [22,46]. For illustration purposes, consider the following message from the QN community to show how codes were assigned to these messages:

YES Cravings will go away!!! It’s hard to believe, I didn't believe it either, but figured a zillion people here said they would and they were right!!! Just hang in, tough it out, stick head in freezer and breath deep and whatever you do, if any chance of slipping might creep up remember the 3 post rule!! Again, the Cravings are going to stop, it’s going to be ok, I promise!!!

This message has embodied an overarching theme of *cravings* where the user who has posted the message is providing advice on how to deal with cravings and is providing specific instructions to the other user in the form of *social support* (communication theme) as to how they can deal with this issue. The user used a *directive* speech act, where the intention was to get one’s peer to remember the three-post rule, where the user is encouraged to make a post if they are craving for a smoke and wait for at least three responses. The user also used an *assertive* speech act, where the user talked about their beliefs associated with cravings, such as “Cravings are going to stop.”

Communication themes and speech act codes were not mutually exclusive and may relate to multiple codes. Two independent researchers were involved in the coding process. Each coder used a qualitative coding schema to independently assign communication theme and speech act categories to a subset of messages by performing line-by-line analysis of every message using directed content analysis techniques [47]. Each message was chosen as the coding unit for analysis. Since we followed a predetermined set of codes to label the peer messages from the two communities, there was no indication for the need for new communication themes or speech acts to capture the content or intent of the communication. Interrater reliability was calculated between coders to ensure objectivity in the coding process using the Cohen kappa (κ; .78 for communication themes and .76 for speech acts) [50].

#### User Engagement and Behavior Characterization

We manually developed an empirical user persona based on users’ engagement levels and behavior profiles. Table 1 provides an overview of the data set characteristics from both communities. The average number of messages posted by QN and ADA community users were 2 (SD=3.37) and 7 (SD=13.73), respectively. Based on this, we divided our users into three engagement levels: high-engagement users (>3 messages for QN and >=8 messages for ADA), medium-engagement users (2-3 messages for QN and 4-7 messages for ADA), and low-engagement users (1 message for QN and 1-3 messages for ADA).

**Table 1:** Data set characteristics.

Characteristics		QN^a^	ADA^b^
Average age of users, years (n=594)		46	—
**Gender distribution (n)**			
	Male	130	—
	Female	463	—
	Not identified	291	—
**User engagement status, n (%)**			
	High	128 (14.5)	31 (21.8)
	Medium	244 (27.6)	29 (20.4)
	Low	512 (57.9)	81 (57.0)
**Tobacco abstinence status (n=254) specific to QN, n (%)**			
	Abstinent users	162 (63.8)	—
	Non-abstinent users	47 (18.5)	—
	Relapsed users	1	—
	Users who started their quit	2	—
	No self-reported status	42 (16.5)	—
**DSM^c^ behavior persona (n=141) specific to ADA, n (%)**			
	Users on medications	—	60 (42.6)
	Users on no medications	—	23 (16.3)
	Users with newly diagnosed T2DM^d^	—	12 (8.5)
	Users with advanced T2DM	—	66 (46.8)
	Users with lifestyle changes	—	36 (25.5)
	Users with no lifestyle changes	—	47 (33.3)
	No self-reported signatures	—	58 (41.1)

^a^QN: QuitNet.

^b^ADA: American Diabetes Association.

^c^DSM: diabetes self-management.

^d^T2DM: type 2 diabetes mellitus.

We also reported their self-reported smoking status by manually analyzing the messages, leveraging the fact that QN community users tend to specify the number of days since they last smoked in their message postings as a form of tradition. From these data, we estimated the user abstinence status for a subset of users (n=254) and identified users as falling into one of the following categories: abstinent (status 1), non-abstinent (status 0), relapsed (status 1 to status 0), users who started their quit recently (status 0 to status 1), and users with no self-reported smoking status.

Further, we constructed a DSM behavior persona for a subset of users (83/141, 58.86%) based on their self-reported forum signature and classified them as follows based on their DSM strategies and diagnostic features: (1) medication versus nonmedication users—the users were differentiated based on whether they managed their diabetes with the help of medications, such as metformin (Glucophage) or insulin, from those who did not use any medications for self-management or had no mention of medication use in their self-reported signatures; (2) users with newly diagnosed T2DM versus users with advanced T2DM—the users were differentiated based on whether they had a diagnosis of diabetes for less than 4 years (2017 or onward) from those that had had a diagnosis of diabetes for over 4 years (earlier than 2017); and (3) incorporation of lifestyle changes versus no incorporation of lifestyle changes—the users were differentiated based on whether they had incorporated lifestyle changes, such as low-carb diets or exercise, into their daily routines from those who did not incorporate such changes or had no such mentions in their self-reported signatures.

### Statistical Analysis

Further, the associations among user behavior and engagement profiles with speech acts and communication themes were evaluated by statistical analysis approaches, such as the chi-square test or the Fisher test, depending on the sample size. We also used the Cramer *V* to assess their correlation strengths. All statistical analyses were performed using the R programming language (using the stats package), and the significance level was *P*<.05.

## Results

### Qualitative Analysis

**Figure 2:**
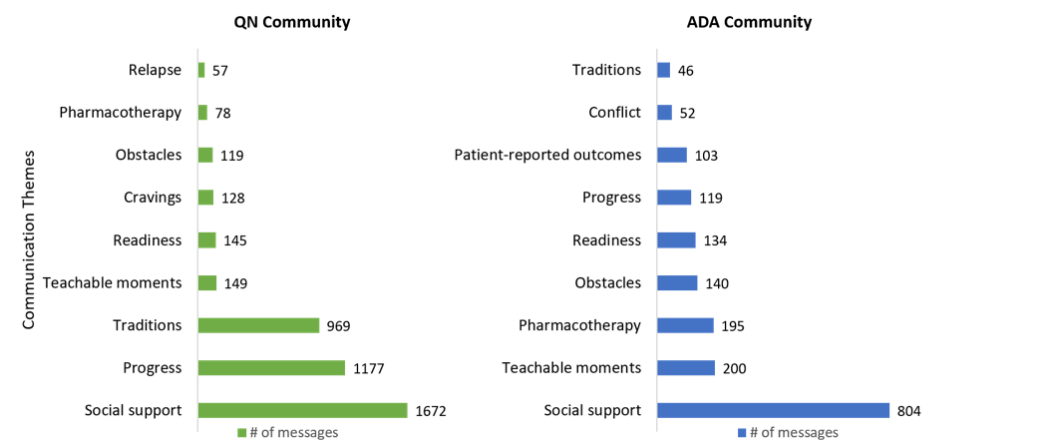
Prevalence of communication themes in QN and ADA communities. ADA: American Diabetes Association; QN: QuitNet.

**Figure 3:**
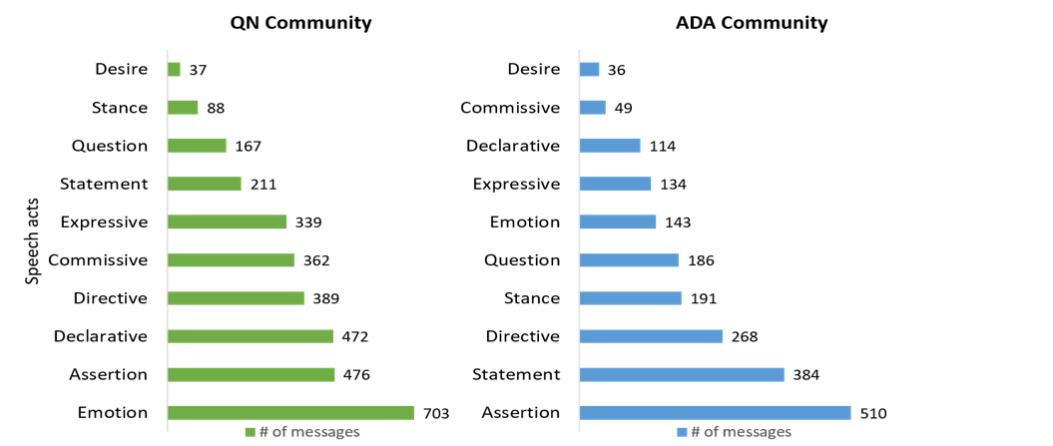
Prevalence of speech acts in QN and ADA communities. ADA: American Diabetes Association; QN: QuitNet.

The qualitative analysis of the 3011 messages exchanged by QN and ADA community users provided insights into the nature of the thematic interest of the community users and the variance in the expression of intentions among them (Figures 2 and 3).

#### QN Community Communication Themes

Community-driven *traditions* were quite popular in the QN community, such as bonfires, which is a virtual event in which users bring their unsmoked cigarettes and throw them into the fire, sharing stats with one another in the form of the number of days since they last smoked and celebratory exchanges upon meeting certain milestones, or pledges, which is a ritual in which users extend their hand to other community users to stay committed to abstinence. Messages describing incentives to not smoke (*teachable moments*), such as improved quality of life (eg, family bonding), better perception of everyday moments (eg, savoring food), reduction in health risks (eg, cancer), were also common in the QN community. The QN community users also posted messages seeking help to fight *cravings* or making a confession to others about their relapse or obstacles that uninspired users to stay quit. *Pharmacotherapy* options in the form of gums and patches that helped users stay away from tobacco were also commonly discussed to help those struggling in staying quit.

#### ADA Community Communication Themes

In the ADA community, medication-related conversations centering around the use of insulin (Lantus), metformin, etc, were quite prevalent (*pharmacotherapy*). Anxiety issues or the inability to manage blood glucose levels within the desired range were the most commonly expressed obstacles among ADA community users (*obstacles*). The ADA community users also shared their *progress* via objective metrics, such as improved A1c values and adherence to healthy diets. *Patient-reported outcomes* were shared by ADA community users, such as higher blood glucose readings caused by beta-blockers.

#### QN Community Communication Intent

The most prevalent intention behind user communication in QN was *emotion*, which emphasizes the nature of providing emotional support to one another through such digital platforms. *Declarative* intentions where QN community users provide objective information in the form of stating their quit dates or days since the last smoke were also common. *Assertions*, such as “It really does get better, people told me that and I thought they were lying, but it really does,” show how an individual’s belief shapes their tobacco cessation–related goals. *Directives*, where QN community users provide support and guidance to one another, and *commissive* intentions, such as pledges or promises, were also prevalent. Some QN community users had *expressive* intentions underlying their communication with other community users, where they tried to convey their feelings about struggles with the quitting process or applauding the achievements of their peers. *Statements* such as “In my first few weeks of this quit I replaced the pack of ciggs that was always in my purse with a stack of index cards” focused on providing information about health practices that helped users stay focused on their health-related goals.

#### ADA Community Communication Intent

In the ADA community, *assertion* speech acts embedded within the messages, such as “Consider blurry vision as a sign of high blood sugar” or “Diet and exercise are the primary tools of defense against diabetes” were commonly present within the peer interactions. There was also a high prevalence of *statements* highlighting health-related practices of community users, such as “Since my diagnosis I have cut down carbs, started exercising and taking metformin with the goal of keeping A1C values close to normal.” *Directives*, such as “Follow up with your primary care physician to get the medications checked” or “Check your blood glucose values at least before every meal in the beginning,” highlighted the presence of peer support and guidance within the community. *Stance* speech acts in the form of “I agree, meds are a source of consternation” or “I disagree with your point” were also prevalent in ADA peer interactions. Many ADA community users looking for guidance from their peers posted their queries or *questions* in the forums. *Declarative* speech acts, such as “I will no longer eat or drink carelessly,” where ADA community users announced objective information regarding their health-related goals, were also common. *Expressive* and *emotion* speech acts were also prevalent in ADA peer interactions to describe their efforts toward DSM or to applaud the achievements of their peers.

**Figure 4:**
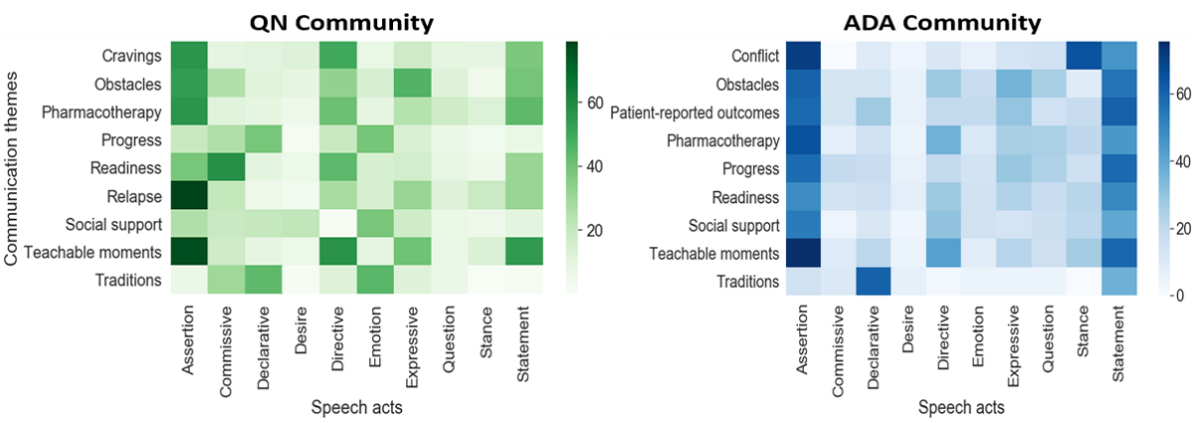
Mapping of communication themes and speech acts captured via peer interactions in the two OHCs. ADA: American Diabetes Association; OHC: online health community; QN: QuitNet.

Figure 4 shows the co-occurrence matrix of communication themes and speech acts for both communities, where the color scale represents the percentage of messages in which a given speech act was present; the x axis represents the speech act categories, and the y axis represents the communication themes categories. In the QN community, there was a high prevalence of *commissive* speech acts in the *readiness* theme, which is expected, since this theme reflects motivations to initiate positive health changes. In addition, there was a high prevalence of *declaratives* in the *traditions* theme, since most of the traditions in the QN community focus on sharing quit statistics with other community users. In the QN community, *directives* were highly prevalent in *teachable moments* and the *cravings* theme. In the ADA community, *directives* were highly prevalent in *pharmacotherapy* theme, where users gave directions to one another on the use of medications, such as insulin and metformin. The *stance* speech act was highly prevalent in ADA messages where users’ opinions were at a *conflict* with other community users, and the *expressive* speech act was commonly present in the *obstacles* theme, where the ADA community users shared their psychological state of dealing with issues such as not managing their diet plan on a day-to-day basis.

### Results From Statistical Analysis

The associations between all categories of speech acts and the five most prevalent categories of communication themes within the QN and ADA communities are shown in Tables 2 and 3, where *P* and V refer to the *P* value and the Cramer *V* value, respectively. In concrete terms, it means that in the QN community, the *emotion* speech act, for example, was often used in the context of the user’s readiness to quit smoking or was often expressed when a user participated in community-specific *traditions* (eg, bonfires, pledges). Similarly, *stance* and *statement* speech acts, for example, were often prevalent in the discussions outlining motivations to quit smoking (*teachable moments*). *Declarative* and *directive* speech acts, for example, were often used in the context of a user’s *progress* with smoking cessation. The strength of association was highest for the *desire* speech act within the *social support* theme (Cramer *V*=.26), followed by the *directive* speech act within the *social support* theme (Cramer *V*=.20) and the *declarative* speech act within the *traditions* theme (Cramer *V*=.15); see Table 2.

**Table 2:** Evaluation of the association between speech acts and communication themes in the QN^a^ community.

	Social support, *P*(*V*)	Progress, *P*(*V*)	Traditions, *P*(*V*)	Teachable moments, *P*(*V*)	Readiness, *P*(*V*)
Assertion	<.001 (0.07)	.04 (0.02)	<.001 (0.13)	<.001 (0.11)	.02 (0.03)
Commissive	<.001 (0.07)	.71	<.001 (0.07)	<.001 (0.06)	<.001 (0.07)
Declarative	<.001 (0.11)	<.001 (0.08)	<.001 (0.15)	<.001 (0.09)	<.001 (0.08)
Desire	<.001 (0.26)	<.001 (0.13)	<.001 (0.12)	<.001 (0.04)	.02 (0.03)
Directive	<.001 (0.20)	<.001 (0.09)	.30	<.001 (0.12)	<.001 (0.10)
Emotion	.14	.73	<.001 (0.08)	<.001 (0.11)	<.001 (0.08)
Expressive	.002 (0.04)	.02 (0.03)	.02 (0.03)	<.001 (0.04)	.18
Question	.11	.51	.94	.10	.68
Stance	<.001 (0.05)	.47	<.001 (0.07)	<.001 (0.04)	.99
Statement	.05 (0.02)	.004 (0.03)	<.001 (0.11)	<.001 (0.14)	<.001 (0.07)

^a^QN: QuitNet.

In the ADA community, for example, the *assertion* speech act was mostly used in the context of a user’s *readiness* to manage lifestyle behaviors that influence diabetes. The *commissive* speech act was often prevalent within the *social support*, *readiness*, and *teachable moments* communication themes. Messages conveying the user’s experiences with hurdles (*teachable moments*) often contained *expressive* and *stance* speech acts. The *social support* theme often had higher prevalence of the *emotion*, *expressive*, and *stance* speech acts (see Table 3).

**Table 3:** Evaluation of the association between speech acts and communication themes in the ADA^a^ community.

	Social support, *P*(*V*)	Progress, *P*(*V*)	Traditions, *P*(*V*)	Teachable moments, *P*(*V*)	Readiness, *P*(*V*)
Assertion	.35	.40	.74	.37	.03 (0.04)
Commissive	<.001 (0.06)	.51	.99	.04 (0.04)	.004 (0.05)
Declarative	.35	.15	.99	.49	.62
Desire	.45	.62	.99	.84	.13
Directive	.22	.51	.89	.07	.24
Emotion	.004 (0.05)	.001 (0.06)	.15	.48	.90
Expressive	<.001 (0.06)	0.74	.14	<.001 (0.07)	.29
Question	.99	.04 (0.04)	.13	.22	.67
Stance	.02 (0.04)	.63	.50	<.001 (0.07)	.84
Statement	.61	.45	.25	.45	.66

^a^ADA: American Diabetes Association.

**Figure 5:**
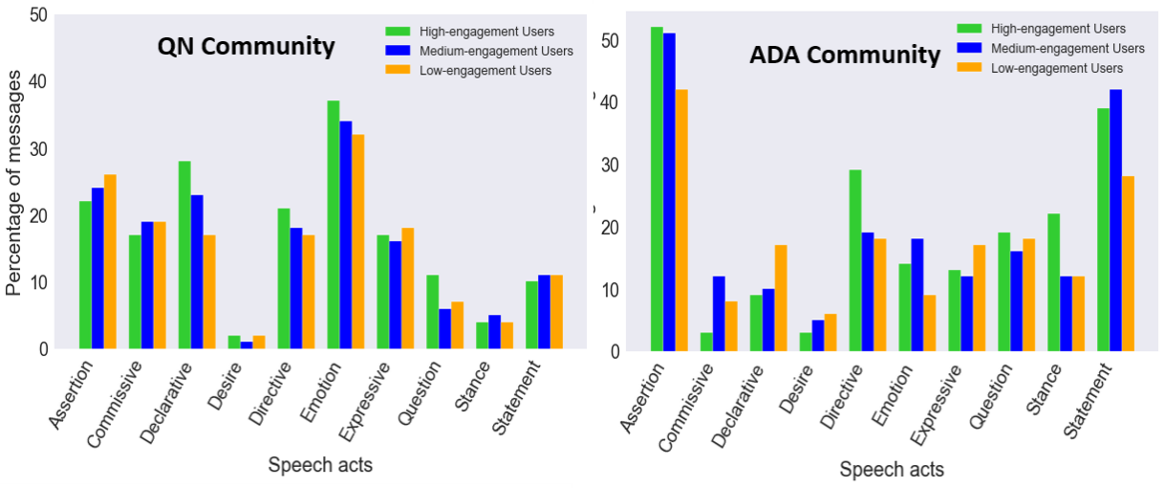
Distribution of speech acts across users’ engagement levels. ADA: American Diabetes Association; QN: QuitNet.

Figure 5 shows the distribution of speech acts across the three user engagement levels for both communities, where the x axis represents the speech act categories and the y axis represents the percentage of messages in which a given speech act was present. In the QN community, low-engagement users had a high prevalence of the *desire* (2%) and *expressive* (18%) speech acts compared to high- or medium-engagement users, which indicates that low-engagement users are willing to incorporate behavior change but somehow need more motivation to engage with other users of the community who have successfully quit. *Emotion* (37%) and *declarative* (28%) speech acts were more frequently expressed by high-engagement users compared to other engagement levels, showing how active users in the QN community play an essential role in providing emotional support to other users in this community. Statistical analysis showed that there is a significant association (*P*<.05) between the overall expression of speech acts and user engagement levels based on the counts of messages expressing different speech acts exchanged by individual user groups, while the overall strength of association is weak (Cramer *V*=.07). Post hoc analysis revealed that there is a significant association (*P*<.05) between the *assertion*, *declarative*, *question*, and *statement* speech acts and the user engagement levels in the QN community (see Table 4).

**Table 4:** Statistical analysis results for users’ engagement levels in the QN^a^ community.

Speech acts	Chi-square or Fisher exact test (*P*)	Cramer *V*
Assertion	.04	0.04
Commissive	.23	—
Declarative	.005	0.06
Desire	.34	—
Directive	.61	—
Emotion	.88	—
Expressive	.47	—
Question	.03	0.05
Stance	.77	—
Statement	<.001	0.13

^a^QN: QuitNet.

Within the ADA community, the low-engagement users expressed the *desire* (6%) and *declarative* (17%) speech acts to a greater extent compared to other engagement levels. The high-engagement users of the ADA community provided *directives* (29%) to other users compared to other engagement levels, which indicates that these users might be able to appropriately guide other community users per users’ concerns. High-engagement users within the ADA community were also quite opinionated as they expressed high levels of *stance* (22%). Statistical analysis showed that there is a significant association (*P*<.05) between the overall expression of speech acts and user engagement levels, while the overall strength of association is weak (Cramer *V*=.13). Post hoc analysis revealed that there is a significant association (*P*<.05) between the *commissive*, *declarative*, *directive*, and *stance* speech acts and the user engagement levels in the ADA community (see Table 5).

**Table 5:** Statistical analysis results for users’ engagement levels in the ADA^a^ community.

Speech acts	Chi-square or Fisher exact test (*P*)	Cramer *V*
Assertion	.80	—
Commissive	<.001	0.11
Declarative	.002	0.08
Desire	.06	—
Directive	.03	0.06
Emotion	.18	—
Expressive	.12	—
Question	.74	—
Stance	.02	0.06
Statement	.28	—

^a^ADA: American Diabetes Association.

**Figure 6:**
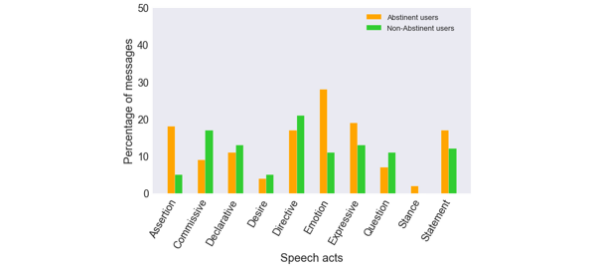
Distribution of speech acts according to the user’s abstinence status in the QN community. QN: QuitNet.

The distribution of different speech acts by the percentage of messages exchanged per the QN community user’s abstinence status is shown in Figure 6, where the x axis represents the speech act categories and the y axis represents the percentage of messages in which a given speech act was present. Abstinent users had different informational needs than non-abstinent users. Their role and context in making contributions differed from those new to the community or in a different stage of behavior change. They had a high prevalence of *emotion* (28%) and *expressive* (19%) speech acts. The other commonly occurring speech acts among abstinent users were *assertion* (18%), *directive* (17%), and *statement* (17%). The most highly prevalent speech act among non-abstinent users was *directive* (21%), and they also expressed themselves with the *commissive* (17%) and *declarative* (13%) speech acts through promises to themselves or the community. The other commonly occurring speech acts among non-abstinent users were *emotion* (13%) and *statement* (12%). The *stance* speech act was only expressed by abstinent users and had low prevalence (2%). The prevalence of the *desire* speech act was comparable across QN community users per their abstinence status—4% for abstinent users and 5% for non-abstinent users.

**Figure 7:**
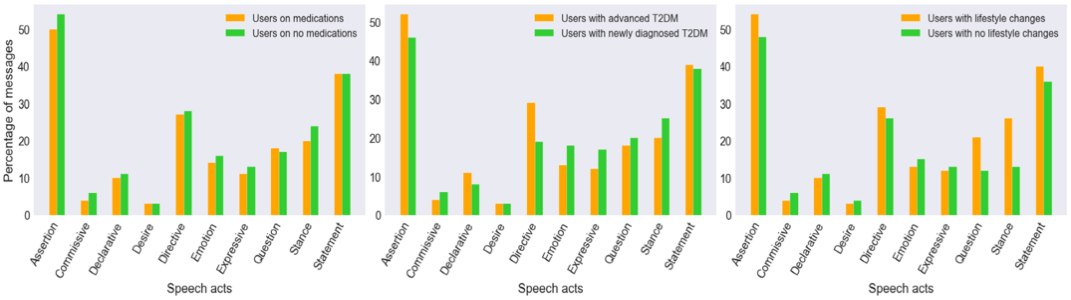
Distribution of speech acts according to the user’s behavior profiles in the ADA community. ADA: American Diabetes Association; T2DM: type 2 diabetes mellitus.

We also examined the distribution of different speech acts by the percentage of messages exchanged per the ADA community users’ medication status, as shown in Figure 7, where the x axis represents the speech act categories and the y axis represents the percentage of messages in which a given speech act was present. The most highly prevalent speech acts for both medication and nonmedication users were *assertion* (50% and 54%, respectively) and *statement* (both 38%). The other commonly occurring speech acts among medication and nonmedication users were *directive* (27% and 28%, respectively), *stance* (20% and 24%, respectively), and *question* (18% and 17%, respectively). Regarding the distribution of different speech acts by the percentage of messages exchanged per ADA community users’ diagnosis status (Figure 7), the most highly prevalent speech acts for newly diagnosed T2DM and advanced T2DM users were *assertion* (46% and 52%, respectively) and *statement* (38% and 39%, respectively). The newly diagnosed T2DM users had a higher prevalence of the *stance* (25%) speech act, while advanced T2DM users had a higher prevalence of the *directive* (29%) speech act. The users who incorporated lifestyle changes provided *directives* (29%) to others and also shared their health practices through the use of *statements* (40%). The users who incorporated lifestyle changes had a high prevalence of the *assertion* (54%), *question* (21%), and *stance* (26%) speech acts in their peer interactions compared with users who had not incorporated lifestyle changes (Figure 7).

Table 6 shows the results of the statistical analysis across user behavior profiles within the two OHCs, where *P* and V refer to the *P* value and the Cramer *V* value, respectively. There was a statistically significant (*P*=.002) association between the expression of speech acts and abstinence status in the QN community. Similarly, there was a statistically significant (*P*=.03) association between the prevalence of various communication themes and abstinence status in the QN community. There was a statistically significant (*P*=.007) association between the expression of speech acts and lifestyle change status in the ADA community. This significance was also noted in the prevalence of communication themes and lifestyle change status within the ADA community (*P*=.001). The relationship strength between speech acts and abstinent status (Cramer *V*=.22) and between speech acts and lifestyle change status (Cramer *V*=.12) was weak. There was no significant relationship between the expression of speech acts and the ADA community users’ medication or diagnosis status. Further analysis is required to identify the specific categories of speech acts that might contribute to these significant relationships.

**Table 6:** Statistical analysis results across user behavior profiles in the two OHCs^a^.

		Chi-square or Fisher exact test (*P*)	Cramer *V*
**Speech acts**			
	QN^b^ (abstinent vs non-abstinent users)	.002	0.22
	ADA^c^ (users on medications vs users on no medications)	.95	—
	ADA (users with newly diagnosed T2DM^d^ vs users with advanced T2DM)	.26	—
	ADA (users with lifestyle changes vs users with no lifestyle changes)	.007	0.12
**Communication themes**			
	QN (abstinent vs non-abstinent users)	.03	0.16
	ADA (users on medications vs users on no medications)	.23	—
	ADA (users with newly diagnosed T2DM vs users with advanced T2DM)	.08	—
	ADA (users with lifestyle changes vs users with no lifestyle changes)	<.001	0.16

^a^OHC: online health community.

^b^QN: QuitNet.

^c^ADA: American Diabetes Association.

^d^T2DM: type 2 diabetes mellitus.

## Discussion

### Principal Results

Our study focused on analyzing the speech acts embedded within the user communications in OHCs using the mixed-methods approach in order to gain an understanding of the factors contributing toward behavior change in the context of two health care domains, tobacco cessation and diabetes self-management (DSM). The study of communication intent to understand and model health-related user communications is not without precedent. For example, earlier research used a similar pragmatics-based discourse analytic framework to investigate the expression of empathy within a T2DM Facebook support group and found that empathy may be expressed and perceived differently depending on the user’s perspectives [51]. Similarly, in our study, although management of T2DM may include promises of behavior change through diet, exercise, and increased adherence to medication regimens, *commissive* speech acts formed only a small fraction of the discussion. Although we used the 10 categories of speech acts from Searle's taxonomy of speech acts, other studies have focused on only stance-taking intentions of complaining or disagreeing [52] or relationship-building through use of the *self-praise* speech act to understand how this speech act instills positive values among the smoking cessation community users [53]. However, it is important to look at all the categories of speech acts in order to characterize what constitutes persuasive communications so that we can better enable individuals to improve their health-related behaviors through high-impact, just-in-time adaptive support.

Another study [54] used a smoking cessation public forum called SmokingisBad to understand how information is shared between initiators/help seekers and respondents/advice givers using the analytical lens of persuasion and interpersonal pragmatics. This study reported how advice givers focus more on the interpersonal side of interaction to provide motivation and support to initiators to become successful quitters [54]. In our study, too, we noticed statistically significant differences in the expression of communication themes as well as speech acts based on the abstinence status of QN community members. The statistically significant association between the speech acts and communication themes discussed among different user behavior subgroups (abstinent vs non-abstinent users, users with lifestyle changes vs no lifestyle changes) corroborates how the intent of peer interactions and social dialogue are unique to an individual’s behavior profile and also differ for a shared collection of communication themes. Abstinent users engaging with a smoking cessation community are likely to have different needs than those seeking support to stop smoking. Non-abstinent users making a promise to themselves or peers through the use of a *commissive* statement (eg, “I will stop smoking tomorrow.”) logically follows. Existing tobacco cessation interventions oftentimes target an individual’s readiness based on their stage of behavior change (as defined by the transtheoretical model of change [55]) and have proven to be effective in helping individuals reduce their nicotine intake [56,57]. In addition to the behavioral stage, our study enables the identification of the cognitive and behavioral state of individuals, as manifested in their peer interactions at a granularity that was not previously explored through incorporation of attributes that describe communication content and intent. The uniqueness of our study lies in understanding the intent of the community users, not just based on one’s conversations with their peers, but also based on their self-reported behavior profiles as well as observed engagement levels, thus offering intrapersonal and interpersonal contexts of psychosocial and behavioral domains crucial to self-management of risky behaviors and chronic health conditions.

Significant associations between speech acts, communication themes, and user engagement within the two OHCs also suggest variances in preferred methods of expression and peer discussions, depending on the engagement levels of users. It is also interesting to note that ADA community users with low engagement had a higher prevalence of the *desire* speech act in their communication with peers, reflecting on their needs to fulfill their self-management goals. However, most ADA community users with high engagement had a high prevalence of the *directive* speech act, which may reflect their ability to provide guidance to their peers, thus emphasizing the role of peer mentors and patient expertise [58]. This provides an additional layer of social complexity that can be harnessed in the design of various digital health tools through the inclusion of advanced interactions in the form of recommendation engines that facilitate meaningful peer and content connections, conversational agents for guided training, etc. Such technological implementations can take into consideration the specific intentions associated with the engagement status of users within an OHC in order to help low-engagement users who need motivation and social support by exposing or recommending them to high-engagement users who have been able to maintain constant engagement using such platforms or to relevant topics based on their thematic interests. The age group of the community users also affects their perceptions and engagement with technology and is another important factor to consider in order to maximize the potential of digital health tools to improve consumer engagement with such platforms to facilitate behavior change [59], which is outside the scope of our analysis due to data constraints associated with our data sets.

In terms of the associations between speech acts and communication themes, all of the speech acts (except *question*) were significantly associated with the *social support* theme within the QN community. Only a few speech acts (*commissive*, *emotion*, *expressive*, and *stance*) were significantly associated with the *social support* theme in the ADA community. As social support is delivered within the full range of speech acts, this indicates robust use of many different subtle forms of transmission, emphasizing how the messages are formed matters. Areas of differentiation in how these ideas are shared (and why it differs in areas such as *progress*, *readiness* for change, and *traditions*) indicate areas where coupling pragmatic action with message content has the potential for impact. The differences observed among online communities to discuss communication themes such as *traditions*, *obstacles*, and *patient-related outcomes* also highlight the distinctive approaches necessary to address independent communities. Previous research has shown that smokers who receive comprehensive cessation counseling over the telephone using techniques that focus on cognitive, emotional, and coping processes tend to have increased acceptance of cravings to smoke [60]. Similarly, in our study, we found that in the QN community, the *directive* speech act was highly prevalent in the *cravings* theme; thus, digital behavior change interventions can benefit from incorporating such additional insights into cravings to improve message crafting and content tailoring capabilities per the user’s needs.

#### Implications for the Design of Digital Health Interventions

Understanding the organic evolution of interaction ingredients in health-related online social conversations facilitates the synthesis of support infrastructure, including virtual chat rooms and digital assistants in health care, and depends on the comparable and adaptive emulation of naturalistic expressions in peer interactions. Such analysis can enable (1) construction of user representations and their information needs and (2) modeling of collective social resilience and human behavior patterns in collaborative endeavors in digital settings. This deeper understanding can result in downstream technologies such as digital therapeutics and virtual coaching agents with real-time naturalistic conversational capabilities. The results of our study indicate that we can use linguistic taxonomies (eg, Searle's taxonomy of speech acts) in order to capture the latent needs of OHC users based on their interactions with their peers, alongside their topics of interest (communication content), which can subsequently result in more responsive and personalized digital health interventions to support behavior change. This can have implications for designing digital care management pathways for individuals and communities based on their self-reported digital behaviors and engagement preferences, thereby enabling individuals to make better choices, and support them in sustaining long-term behavior change, thus ultimately improving their quality of life.

### Limitations

Our study is not without limitations. Although QN and ADA messages were selected at random, the relatively small and uneven sample sizes may have resulted in inaccurate representations of the overall prevalence of speech acts and thematic emphasis. However, our sample of 3011 messages using qualitative research methods is appropriate for the study objectives. Even though we used empirically sound and reliable methods for coding the peer conversations, our analysis might be limited by subjective bias in the annotation process that is inherent in human coding in qualitative research [61]. Although we extracted the behavior profiles of the users who had self-reported their abstinence status within the QN forum messages and those who had created self-reported signatures within the ADA community, such extraction may be affected by incomplete or inaccurate self-report accounts and as such may not be representative of the general population. Moreover, our analysis did not consider sociodemographic and cultural factors, which can also result in differences in expressions. Our analysis was primarily focused on text-based user communications and did not consider other aspects of online user behavior within an OHC (eg, likes, dislikes, shares), which can also provide additional insights. OHCs can also be used for spreading health-related misinformation [62], which can have a negative influence on such behaviors. However, we did not consider the manifestation of misinformation as a separate theme within our data sets, which should be formalized through content-flagging mechanisms in future studies. The timeline of the data sets used in the study is not recent (2000-2015 for QN and 2015-2018 for ADA). We will attempt to obtain current data sets in future studies; however, we believe the findings still hold since the basic tenet of the social interactions remains the same. Future work should consider coding more messages in order to ensure category saturation for speech acts and communication themes so as to curate a balanced annotated data set. Our efforts to develop semiautomated methods to scale up the application of communication themes and speech act labels to large-scale data sets using deep learning models (eg, convolutional neural networks [CNNs]) [63], bidirectional encoder representations from transformers (BERT) [64]), and social influence models [65-67] are underway.

### Conclusions

Digital health platforms, specifically OHCs, provide unprecedented opportunities for researchers to transform into empathetic listeners of people’s endeavors, from small-scale, 1-day-at-a-time to large-scale life-consuming behavior modifications. Such listening capabilities will help us glean the needs of individuals to initiate and sustain positive health changes. This paper proposes an analytical framework to enable deep social listening capabilities to uncover the unmet needs of individuals, thus laying the foundation for next-generation technology innovation efforts. Our efforts to overlay the communication intent implicit in peer interactions alongside content-specific theory-linked characterizations of social media discourse have provided insights into topic diversity and latent interactions of users within these topics. Such content-specific and intent-sensitive methodological framework can inform the development of cognitively enabled big data analytics and machine learning models that better harvest the digital footprint of social media users.

## Appendix

Multimedia Appendix 1 Qualitative coding schema for speech acts.

Multimedia Appendix 2 Qualitative coding schema for communication themes.

## Abbreviations

ADA: American Diabetes Association
DSM: diabetes self-management
OHC: online health community
QN: QuitNet
T1DM: type 1 diabetes mellitus
T2DM: type 2 diabetes mellitus
