# Supplementary material for: Pragmatics to Reveal Intent in Social Media Peer Interactions: Mixed Methods Study
Source: J Med Internet Res. 2021 Nov 17;23(11):e32167. doi: 10.2196/32167 (PMC8663565; doi:10.2196/32167)
Supplement: Multimedia Appendix 1 [file jmir_v23i11e32167_app1.docx]

Multimedia Appendix 1. Qualitative coding schema for Speech Acts.

| Speech Acts | Example Messages |
| --- | --- |
| *Assertion:* stating something definitively using one’s belief, including prediction, judgement, guess | QN: “Now we have all quit smoking, we will have more days of our lives back just to celebrate.”  ADA: “Stress can have a huge impact on your numbers.  Even a single stressful day can raise my numbers significantly and I have had longer periods of stress that I know upped my A1C” |
| *Commissive:* committing the speaker to some future action | QN: “I've set tomorrow as my quit date and have been planning for tomorrow for a couple of weeks”  ADA: “I pledge to swap desserts for fresh fruit from now on” |
| *Declarative:* announcing objective information | QN: “No more missing life for a dagum smoke!”  ADA: “I know the benefit of testing often and so I am still doing it, even if I am not advised to.” |
| *Desire:* describing of plan to do something to get to a future state | QN: “I want to smoke soooo bad”  ADA: I wish someone had told me to get a meter, years ago, before I was having any evident trouble, and certainly as my numbers became "borderline"” |
| *Directive:* getting the hearer to do something | QN: “Just let it fly and get out all the stress and frustrations of the day!”  ADA: “You have to test more often than that, and you have to be committed to using your test results to alter what you do in terms of eating and exercise” |
| *Emotion:* expressing feelings or emotions | QN: “OMG I can’t believe that I have not smoked for almost 2 months - its huge”  ADA: “I appreciate the (PPG testing) suggestion, and it has been heard.” |
| *Expressive:* expressing the speaker’s psychological state | QN: “Congratulations on a fabulous 7 months”  ADA: “I'm so down in the dumps and discouraged. Today's it's bee up all day (140s) and I've eaten very so little. Help!” |
| *Question:* asks for information | QN: “has anyone managed to get addicted to the inhaler instead of smoking??”  ADA: “Can Diabetes make you lost a lot of weight?” |
| *Stance:* agrees or disagrees with a peer | QN: “I see both sides, but agree with Clear Colors. I've relapsed so many times I can't count them, but I am here now and working my plan”  ADA: “I agree that our bg meters are not exact and show variation and that our blood glucose concentrations vary.” |
| *Statement:* description or explanation of a health practice | QN: “Since quitting smoking cooking has become a new hobby for me...never could cook or cared much to because of course you cannot smoke around the food”  ADA: “I'm still on a prescribed protocol eating 60 to 75 carbs per meal, most meals, call it 200 per day, unless I just happen to have a Cobb salad or something at lunch that totals just 20 or so. Still my lowest day since I've been on this, has probably been 150 carbs.” |

QN: QuitNet

ADA: American Diabetes Association
