# Supplementary material for: Pragmatics to Reveal Intent in Social Media Peer Interactions: Mixed Methods Study
Source: J Med Internet Res. 2021 Nov 17;23(11):e32167. doi: 10.2196/32167 (PMC8663565; doi:10.2196/32167)
Supplement: Multimedia Appendix 2 [file jmir_v23i11e32167_app2.docx]

Multimedia Appendix 2. Qualitative coding schema for Communication Themes.

| Communication Themes | Example Messages |
| --- | --- |
| *Social support:* Messages where the content reflects the elements of *praise, advice, empathy*, and *guidance* | QN: “You are a Q hero! Congratulations, you've worked hard for this and I'm delighted to celebrate with you.”  ADA: “congratulations! Now you have to keep it up for a lifetime. Hope you stick around here.” |
| *Progress:* Messages in which users communicate their progress based on objective health measures (e.g. *days since last smoke*) | QN: “5 months for me today. I love being a non-smoker.”  ADA: “95 this morning, my first double digit result in 2 weeks!” |
| *Traditions:* Messages that focus on community-specific rituals such as *bonfires, pledges, games*. | QN: “Good evening wonderful Qsters and welcome to the Monday Night Bonfire on Q Island!! I’m thrilled and honored to be your hostess tonight”  ADA: “will be walking every morning with trek poles getting ready for walk l-3 miles in” |
| *Teachable moments:* Messages that describe incentives to make positive health changes | QN: “it is so nice not to be going nuts over having no lighter/ciggies/place to smoke, it's great to have that freedom!”  ADA: “I switched to low-carbs 5 years ago, my diabetes was making me a bad mom low energy all day” |
| *Readiness:* Messages that inspire to initiate positive health changes | QN: “No more excuses, no more stinking thinking, get tough, get real”  ADA: “What started out to be a rather large challenge (no rice, no bread, no pasta, no potatoes) to no big deal. I can handle this.” |
| *Cravings:* Messages hat capture real-time expressions of the urges to deviate from planned health behaviors (e.g. *to light up a cigarette*) | QN: “There HAS to be other little "Tricks" people use. C'mon people...let us all in on your secrets... what DO you do when a crave hits you???” |
| *Obstacles:* Messages focusing on hurdles to planned health practices (e.g. *weight gain with quitting tobacco*) | QN: “I think that the hardest part of this is not knowing whether my symptoms are the new normal or if they will pass once withdrawal is over.”  ADA: “I'm starting to pass out, I don't feel good lay down. I can't study when sugar is so low.” |
| *Pharmacotherapy:* Messages with explicit discussions on various pharmacotherapy options (e.g. *Chantix, Zyban*) | QN: “The Surgeon General's report cites success with the combination of Zyban and NRT”  ADA: “Armour Thyroid is just another option to explore. Purity was not mentioned.” |
| *Relapse:* Messages with descriptions of relapse reasons and confessions | QN: “I hate myself, I slipped again. I lighted the nicodemon”  ADA: “I was a bad boy last night. So today its 273.” |
| *Conflict* | ADA: “I can see what you mean, but I don't think all of the conclusions you've reached are necessarily true/logical.” |
| *Patient-reported outcomes* | ADA: “I started the Trulicity on Tues and have been ultra-careful with my diet and have started walking in the morn etc. My morning b.s. is around 200 and after breakfast even higher.” |

QN: QuitNet

ADA: American Diabetes Association
